# Location, location, location…site-specific GPCR phosphorylation offers a mechanism for cell-type-specific signalling

**DOI:** 10.1016/j.tips.2008.05.006

**Published:** 2008-08

**Authors:** Andrew B. Tobin, Adrian J. Butcher, Kok Choi Kong

**Affiliations:** Department of Cell Physiology and Pharmacology, Hodgkin Building, Lancaster Road, University of Leicester, LE1 9HN, UK

## Abstract

It is now established that most of the ∼800 G-protein-coupled receptors (GPCRs) are regulated by phosphorylation in a process that results in the recruitment of arrestins, leading to receptor desensitization and the activation of arrestin-dependent processes. This generalized view of GPCR regulation, however, does not provide an adequate mechanism for the control of tissue-specific GPCR signalling. Here, we review the evidence that GPCR phosphorylation is, in fact, a flexible and dynamic regulatory process in which GPCRs are phosphorylated in a unique manner that is associated with the cell type in which the receptor is expressed. In this scenario, phosphorylation offers a mechanism of regulating the signalling outcome of GPCRs that can be tailored to meet a specific physiological role.

## Introduction

The earliest studies of GPCR phosphorylation determined the involvement of more than one protein kinase mediating multi-site phosphorylation [1,2]. In these studies, the β_2_-adrenoceptor was shown to be phosphorylated at low levels of receptor occupancy by protein kinase (PK) A in a process that resulted in the phosphorylation of sites in the third intracellular loop and C-terminal tail of both the agonist-occupied and -unoccupied receptor. However, at high agonist occupancy the receptor was phosphorylated additionally by members of the G-protein-coupled-receptor kinase (GRK) family at sites in the C-terminal tail that were distinct from the PKA sites [3–5]. The key features of these studies were that, first, very different protein kinases are in operation that have very different mechanisms of activation. Second, that these protein kinases phosphorylate distinct sites on the receptor with different kinetics. Third, the signalling consequences of these phosphorylation events have some overlapping features (e.g. they both result in receptor desensitization) but they also have unique features (e.g. phosphorylation mediated by the GRKs, but not that by PKA, mediates receptor internalization and arrestin recruitment) [2].

Hence, these studies carried out on the β_2_-adrenoceptor over a decade ago provided clear evidence for differential phosphorylation mediating differential signalling outcomes. However, the possibility that this process might be employed in a tissue-specific manner to tailor specific signalling outcomes of GPCR subtypes in physiological cell types has, until recently, been largely overlooked [6]. This can be attributed to the desire to determine a ‘unifying hypothesis’ that explains the regulation of all GPCR subtypes regardless of the cell type in which they are expressed. Thus, all but a few GPCRs are considered to be phosphorylated by one or more of the family of seven GRKs (GRK1 through to GRK7) in a process that results in the uncoupling of the receptor from its G protein and the recruitment of one of four subtypes of arrestin [7,8]. Whereas this process is undoubtedly in operation, it belies the complex nature of the phosphorylation of GPCRs. It also does not offer a mechanism by which GPCRs can be regulated in a tissue-specific maner. It seems likely that the same receptor expressed in different tissue types will be regulated in a manner that reflects the receptors physiological role. Therefore, a M_2_ muscarinic receptor, for example, expressed on cardiac pacemaker cells with control over contractile rates of the heart [9] would be expected to have different regulatory features from the same receptor expressed in prejunctional parasympathetic neurones in the lung [10].

Recent studies have indicated that the process of receptor phosphorylation and arrestin recruitment does not always conform to the simplified model described but might, in fact, be heterogeneous, offering a broad range of signalling outcomes.

## The heterogeneity of receptor–arrestin interaction

It is widely accepted that the primary function of receptor phosphorylation is to promote the recruitment of arrestin to the activated receptor [7]. Whereas this process has been clearly resolved in the case of rhodopsin interaction with arrestin1 [11], it is far harder to conceptionalize how the diverse nature of phosphorylation on the ∼800 non-visual GPCRs, which show very little sequence homology in their intracellular domains [12], results in the same outcome, namely arrestin recruitment. This conundrum has led some to suggest that phosphorylation simply increases bulk negative charge on the intracellular surface of the receptor and it is this that is important in the recruitment of arrestin rather than the precise nature of the of phosphorylation site [11].

However, there is considerable variation in the ability of receptors to interact with arrestins. Despite the fact that the β_2_-adrenoceptor is highly phosphorylated after agonist stimulation, this receptor interacts with low affinity with arrestins and shows preferential binding to arrestin3 over arrestin2; these are characteristics described as attributable to class A receptors [13]. This contrasts with class B receptors (such as the V_2_ vasopressin receptor), which form high-affinity complexes with both arrestin2 and arrestin3 that are sufficiently stable to be maintained as the receptor traffics to intracellular compartments after agonist occupation [13]. The functional impact of this heterogeneity might be profound because it is now clear that the arrestin isotypes have distinct interacting partners and signalling roles. In the case of the angiotensin AT_1A_ receptor, which is a class B receptor that recruits both arrestin2 and arrestin3, it was found that only arrestin3 supports G-protein-independent extracellular signal-related kinase (ERK-1/2) activation. By contrast, both arrestin isotypes support AT_1A_-receptor desensitization equally [14]. Thus, the signalling outcome from arrestin recruitment to this particular receptor will be determined by the balance of the competition between arrestin2 and arrestin3 for interaction with the phosphorylated receptor. Furthermore, recent proteomic analysis of the interacting partners with the arrestins demonstrated that 71 proteins selectively interacted with arrestin2, whereas a staggering 164 were able to interact selectively with arrestin3 [15]. It is possibly too early to determine the functional significance of these findings but it, nevertheless, shows the potential differential cellular role of the two arrestin subtypes and the impact that differential arrestin recruitment might have in receptor signalling.

The heterogeneity in the relationship between receptor phosphorylation and arrestin recruitment extends beyond class A and class B receptors to extreme cases in which receptor phosphorylation seems to play no part in arrestin recruitment. Protease-activated receptor (PAR)-1 and substance P and leukotriene B4 receptors are examples of receptors whereby arrestin binding is independent of receptor phosphorylation [16–18]. That this phenomenon is more widespread is illustrated by studies on the orexin OX_1_ and PAR-2 receptors, in which removal of putative phospho-acceptor sites has no significant effect on the ability of these receptors to interact with arrestins [19,20].

An explanation for this heterogeneity lies in the fact that receptor–arrestin interaction consists of two distinct components [11]. The first is the interaction of arrestin with the agonist-occupied conformation of the receptor. This occurs via the ‘activation’ sensor, which is proposed to consist of multiple points of contact between the agonist-occupied receptor and the concave surface of arrestin [21]. The second component is the interaction of phosphates contained on the phosphorylated receptor with the ‘phosphate’ sensor situated in the polar core of arrestin [22]. Disruption of the salt bridge at the polar core results in conformational changes in arrestin that promote a high-affinity receptor–arrestin interaction [11]. In the case of rhodopsin–arrestin1, the co-operative binding shown between the activation and phosphate sensors increases the affinity of arrestin1 for the receptor by an order of magnitude [23]. By contrast, despite the hyperphosphorylated nature of the agonist-occupied non-visual GPCRs, the contribution made by the interaction with the phosphate sensor on arrestin2 or arrestin3 is modest, often only resulting in a 2–3 fold increase in affinity [11,23]. However, where phosphorylation seems to have no role in arrestin recruitment, it seems that the interaction of the active conformation of the receptor with the arrestin-activation sensor is itself sufficient to provide a functional receptor–arrestin complex.

In some receptor subtypes the need for phosphorylation to promote arrestin recruitment has been eliminated by the presence of negatively charged residues that function as phosphomimetics. An acidic cluster in the C-terminal tail of the chemokine receptor D6 [24] and an aspartate in the third intracellular loop of the luteinizing hormone and choriogonadotropin receptor [25] are thought to interact with the phosphate sensor in arrestin and thereby mediate phosphorylation-independent arrestin interaction. Interestingly, despite the absence of a requirement for phosphorylation in the recruitment of arrestin these receptors are still phosphorylated [26,27]. The role of receptor phosphorylation in these instances is unknown but it is likely to extend beyond arrestin recruitment.

## Can GRKs regulate differential signalling via differential phosphorylation?

The GRKs have a broad substrate specificity favouring serine and threonine residues contained within acidic-rich regions and share many GPCR substrates [12]. It is, however, not clear whether the various members of the GRK family can phosphorylate different sites on the agonist-occupied receptor.

This question has recently been addressed in an elegant study that used fluorescence resonance energy transfer between a cyan-fluorescent-protein-tagged β_2_-adrenoceptor and yellow-fluorescent-protein-tagged arrestin3 to visualize, in real time, the two components of arrestin recruitment; namely, a rapid phosphorylation-independent association followed by a slower phosphorylation-dependent phase [28]. Surprisingly, preventing GRK-mediated phosphorylation had no effect on the overall recruitment of arrestin3 to the receptor. Rather, it was the kinetics of the slow phase of arrestin recruitment that was regulated by GRK phosphorylation [28]. The subtlety of the role of each of the GRKs was revealed by small interfering RNA (siRNA) silencing of each GRK subtype –it was shown that GRK2 mediates the majority of β_2_-adrenoceptor phosphorylation, but it is GRK6 activity that has the most impact on the rate of arrestin recruitment [28]. Interestingly, GRK6 is the only one of the GRKs to phosphorylate the receptor at residues 355 and 356 on the C-terminal tail. These studies, therefore, raise the possibility that the GRKs might be able to differentially phosphorylate the β_2_-adrenoceptor with the result of determining different rates of arrestin recruitment.

Not only might the rates of arrestin recruitment be dependent on the subtype of GRK involved in receptor phosphorylation but, judging by studies on the V_2_ vasopressin and AT_1A_ angiotensin receptors, so might the signalling function of arrestin [29,30]. In the case of these receptors, GRK2 and GRK3 are again the primary GRK subtypes responsible for agonist-dependent phosphorylation. However, inhibition of GRK2 and GRK3 activity has little effect on arrestin-mediated ERK-1/2. This contrasts with inhibition of GRK5 and GRK6 activity, which has no significant effect on receptor phosphorylation but, nevertheless, results in a reduction in arrestin-dependent ERK-1/2 activation. The argument posed by the authors was that GRK2 and GRK3 are able to phosphorylate sites on the receptor that confer a conformation on arrestin that is not conducive to the activation of the ERK-1/2 pathway. By contrast, GRK5- and GRK6-mediated phosphorylation results in a conformation of arrestin that promotes activation of ERK-1/2 signalling [29,30]. A similar distinction between the signalling properties of receptor phosphorylation via GRK2/3 and GRK5/6 has recently been reported for the β_2_-adrenoceptor [31] and follicle-stimulating hormone (FSH) receptor [32].

The notion that arrestin might adopt a variety of conformations depending on the type of phosphorylation sites on the receptor is supported by evidence that the nature of the complex formed between arrestin1 and rhodopsin depends on the number of sites on rhodopsin that are phosphorylated [33]. Furthermore, in the case of the N-formylpeptide receptor, arrestin binding is influenced by the phosphorylation status within two serine and theronine clusters. Here, phosphorylation within one cluster mediates a different arrestin-dependent effect on receptor function compared with that observed after phosphorylation of the other cluster [34].

## The complexity of GPCR phosphorylation

Numerous studies using a myriad of techniques such as mass spectrometry [35], phosphopeptide mapping [36,37], phosphospecific antibodies [5,38] and mutagenesis [4,32,39] have determined that GPCRs are multiply phosphorylated after agonist occupation. These phosphorylation events occur largely at the C-terminal tail or third intracellular loops of receptors [4,32,39,40], but can also occur within regions of the first and second intracellular loops [41,42]. Whereas the majority of receptor phospho-acceptor sites are in serine-and-threonine-rich regions of the intracellular domains, there is also evidence that GPCRs are phosphorylated on tyrosine residues [43,44] in a manner that can, in some instances, generate classical phosphotyrosine protein-interaction motifs [45]. It is now clear that this diversity of phosphorylation is mediated by receptor kinases that extend beyond the GRKs, PKA and PKC to include protein kinase CK2 [37,46], CK1α [47–49], PKB (also known as Akt) [50–53] and receptor tyrosine kinases [52]. In the case of the β_1_- and β_2_-adrenoceptors, for example, these receptor subtypes are phosphorylated by members of the GRK family, PKA and PKC [1,12,54], but in addition these receptors have been demonstrated to be phosphorylated by the insulin receptor, insulin-like receptor and PKB [52]. Hence, GPCRs can be phosphorylated by multiple protein kinases at multiple sites throughout their intracellular domains in a manner that can be considered as providing a broad dynamic range in which receptor signalling can be modulated.

Thus, one mechanism by which cell-type-specific regulation of receptor signalling could be achieved is by cells employing a restricted complement of receptor kinases in receptor phosphorylation (Box 1). This could be achieved either by differential expression, activation or scaffolding of the receptor kinases.

The potential to regulate the signalling outcome by differential phosphorylation is illustrated by studies on the M_3_ muscarinic receptor, which is phosphorylated not only by GRK2 and GRK6 [49,55,56] but also by CK1α [47,49] and protein kinase CK2 [37]. Tryptic phosphopeptide maps have revealed that these protein kinases can phosphorylate distinct sites on the third intracellular loop of the M_3_ muscarinic receptor [37]. siRNA knockdown of CK2 results in an ∼72% reduction in receptor phosphorylation; however, this does not affect the internalization or coupling of the receptor to the ERK-1/2 pathway [37]. By contrast, inhibition of CK2 activity results in enhanced Jun-kinase signalling [37]. These results demonstrate that phosphorylation of the M_3_ muscarinic receptor by CK2 has a specific impact on the signalling of the receptor to the Jun-kinase pathway but no impact on other phosphorylation-dependent pathways. These data are consistent with earlier studies that established that GRK-mediated phosphorylation of the M_3_ muscarinic receptor is responsible for receptor internalization and desensitization, and that CK1α activity regulates the coupling of the receptor to calcium signalling [49] and the ERK-1/2 pathway [48,55,57]. Thus, in the case of the M_3_ muscarinic receptor, there is evidence that site-specific phosphorylation, by a range of receptor kinases, can result in specific signalling outcomes that are dependent on the protein kinase employed in the phosphorylation [37,49].

This conclusion can also be drawn from an elegant study of the somatostatin sst_2A_ receptor, in which, despite the fact that the majority of agonist-regulated phosphorylation occurs at serine residues (∼80%), it is ‘minority’ phosphorylation on threonine residues throughout the third intracellular loop and C-terminal tail that is important for efficient arrestin recruitment and internalization [40]. Desensitization of the sst_2A_ receptor, however, requires both theronine and serine phospho-acceptor sites [40]. Thus, in a manner similar to that described for the M_3_ muscarinic receptor, differential phosphorylation of the sst_2A_ receptor results in differential signalling outcomes.

## Physiological consequences of differential GPCR phosphorylation

**Figure 1:**
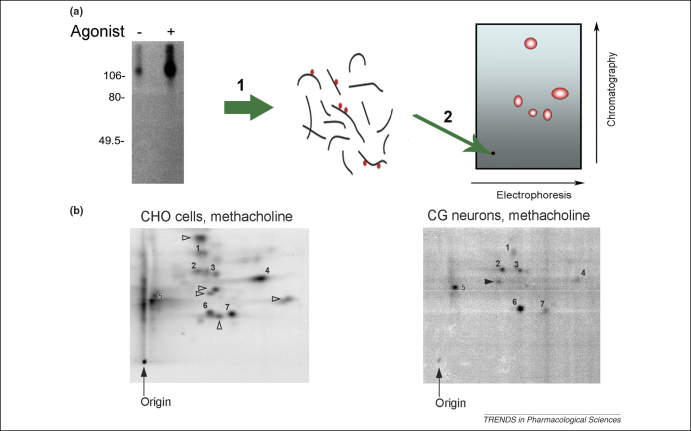
Differential phosphorylation of the M_3_ muscarinic receptor in CHO cells and cerebellar granule neurones. **(a)** Schematic representation of the generation of a receptor phospho-peptide map that reveals a specific pattern of phospho-peptides referred to here as a phosphorylation signature. The receptor (e.g. M_3_ muscarinic) is immunoprecipitated from cells labelled with [^32^P]-orthophosphate using a receptor-specific antibody. (1) The radiolabelled receptor is excised from the SDS–PAGE gel and the gel slice is subjected to tryptic digest. (2) The resulting tryptic peptides are spotted onto a cellulose plate and the plate is subjected to electrophoresis in one dimension and to ascending chromatography in the other dimension (for details, see Ref. [37]). **(b)** Shown are two phospho-peptide maps of the M_3_ muscarinic receptor. (i) The map from the receptor isolated from CHO cells transfected with the mouse M_3_ muscarinic receptor. (ii) The map generated from the M_3_ muscarinic receptor endogenously expressed in cerebellar granule neurones (CGNs). It can be seen that there are phospho-peptides that are common to the two maps (labelled with numbers) in addition to phospho-peptides that are specific either to receptors isolated from CHO cells (open arrows) or from receptors isolated from CGNs (closed arrow). This experiment demonstrates that the M_3_ muscarinic receptor does have a cell–type-specific phosphorylation signature. Figure reproduced, with permission, from Ref. [37].

If the notion that differential receptor phosphorylation is employed to mediate cell-type-specific receptor signalling is true, it should be possible to detect cell-type-specific receptor phosphorylation. The low expression levels of GPCRs have made this very challenging, but recent studies have determined cell-type-specific phosphorylation of the M_3_ muscarinic receptor [37]. In these studies, tryptic-phosphopeptide maps from M_3_ muscarinic receptors expressed in Chinese hamster ovary (CHO) cells and natively expressed in cerebellar granule neurones have produced a ‘phosphorylation signature’ that demonstrates that there are common sites of phosphorylation of the receptor between these cell types but, importantly, there are also sites that are specifically phosphorylated depending on the cell type [37] (Figure 1). These studies, therefore, demonstrated that the M_3_ muscarinic receptor is phosphorylated in a cell-type-specific manner. The phosphorylation signature might encode signalling properties on the receptor where common phosphorylation events mediate common regulatory features such as arrestin recruitment and internalization and where cell-type-specific events mediate specific signalling functions related to the specialized physiological role of the receptor.

This tissue-specific phosphorylation code might be the result of the tissue-specific employment of receptor kinases to provide a unique phosphorylation signature on the receptor (Box 1).

That such a mechanism might exist is illustrated by work on knockout animals lacking the genes encoding members of the GRK family, in which it has been established that GRKs can be differentially employed in the regulation of muscarinic receptor subtypes in the heart and lung. In these studies, the postjunctional M_2_ muscarinic receptors on airway smooth muscle were seen to be desensitized by GRK5 [58]. However, neither prejunctional M_2_ receptors on parasympathetic neurones innervating the lung nor M_2_ muscarinic receptors that are expressed in the heart are regulated by GRK5 [58]. Furthermore, despite the fact that M_2_ muscarinic receptors present on the airway smooth-muscle cells are regulated by GRK5, the M_3_ muscarinic receptors on the same smooth-muscle cells seem not to be influenced by GRK5 activity [58]. These *in vivo* studies provide evidence, at least in the case of GRK5, that GPCR phosphorylation shows both subtype specificity (i.e. M_2_ versus M_3_) and tissue specificity (i.e. airway smooth muscle versus cardiac tissue).

## Potential mechanisms that generate differential GPCR phosphorylation

How receptor kinases, which are usually ubiquitously expressed (such as GRK2, GRK5 and CK2), are regulated in a manner that enables differential phosphorylation of receptor subtypes is uncertain. It is, however, likely that the relative expression levels of the kinases together with specialized scaffolding proteins play an important part. Certainly, the contribution made by the various members of the GRK family to β_2_-adrenoceptor phosphorylation is dependent on their relative expression levels and this, in turn, influences the kinetics of arrestin recruitment to the receptor [28]. Although studies of the comparative expression levels of the GRKs and arrestins in native tissues are limited, there is evidence of changes in the expression levels of these proteins during neuronal development [59] and in neuropathology [60].

The role of scaffolding proteins in coordinating protein phosphorylation is now well established [61]. The fact that scaffolding has an important role in GPCR phosphorylation is illustrated by the coordinated phosphorylation of the β_1_-adrenoceptor by PKA through scaffolding provided by the A-kinase-anchoring protein AKAP-79 [62,63]. It is likely, therefore, that differential scaffolding of receptors with receptor kinases, together with differences in the relative expression levels of the receptor kinases, will contribute to the mechanism by which the same GPCR subtype can be differentially phosphorylated in different tissues.

## Concluding remarks

A common feature of each GPCR subtype is that they can couple to a plethora of signalling pathways. In a physiological setting, however, these receptors are likely to activate just a small complement of their full signalling repertoire in a manner that reflects their physiological role. The process by which receptors mediate cell-type-specific signalling will undoubtedly involve several factors including cell-type-specific expression of downstream signalling components (e.g. cyclases and phospholipases) and the nature of the receptor-signalling complex [64].

In this review, we have considered the possibility that GPCRs are able to be phosphorylated in a cell-type-specific manner and that the site-specific phosphorylation of GPCRs can regulate specific signalling outcomes (Table 1). Hence, it is possible that one mechanism employed by a cell to tailor GPCR signalling to suit a specific physiological role might be receptor phosphorylation, which seems to have sufficient diversity and dynamic range to accommodate the numerous physiological demands placed on each GPCR subtype.

## Figures and Tables

**Figure I:**
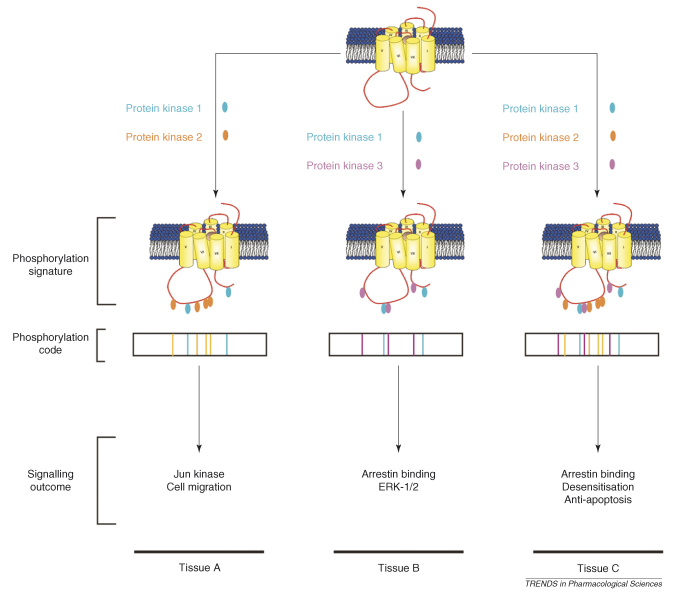
Cell-type-specific phosphorylation of a GPCR by three different protein kinases.

**Table 1:** Examples of receptor site-specific phosphorylation and functional correlates

GPCR	Possible phosphorylation sites and motif	Region	Kinase	Cell type	Function	Refs
Angiotensin type II AT_1A_	Thr332, Ser335, Thr336, Ser338	C-terminal tail	ND	CHO^a^	Internalization	[65]
β_1_-adrenoceptor	Ser412	3i-loop	PKB (Akt)	CHO, cardiac myocytes	Internalization	[66]
β_2_-adrenoceptor	Thr384, Ser396, Ser401, Ser407	C terminus	GRK2	*In vitro*	ND	[67]
Thr384, Thr393, Ser396, Ser401, Ser407, Ser411		GRK5	*In vitro*	ND	
Ser355, Ser356, Ser364	C terminus	GRK	HEK293	Desensitization	[4]
Ser261, Ser262 (also Ser345, Ser346)	3i-loop and/or C-terminal tail	PKA	COS-7 and/or HEK293	Desensitization	[4]
Tyr350 (also Tyr354, Tyr364)	C terminus	Insulin receptor	DDT_1_MF2	Association with Grb2 and internalization, inhibition of cAMP production	[45]
Tyr132, Tyr141	2i-loop	IGF-1 receptor	DDT_1_MF2	Internalization	[45]
Follitropin receptor (FSH)	Thr369, Ser370, Ser371, Thr376	1i-loop	ND	HEK293	Internalization. Contributes to desensitization	[42]
Thr536, Thr541, Ser544, Ser545, Ser546, Ser547, Thr549	3i-loop	ND	HEK293	Contributes to desensitization	[32]
Thr638, Thr640, Ser641, Ser643, Thr644	C terminus	ND	HEK293	Internalization and desensitization	[32]
M_2_ muscarinic	N-cluster (Ser286–Ser290)	3i-loop	ND	HEK293	Internalization	[68]
C-cluster (Thr307–Ser311)	3i-loop	ND	HEK293	Internalization and desensitization	
M_3_ muscarinic	Lys370–Ser425	3i-loop	CK1α	CHO	ERK-1/2 activation	[48]
Ser351-Arg-Ser-Ser-Asp-Glu-Glu-Asp356	3i-loop	CK2	CHO	JNK kinase activation	[37]
S1P_1_ (EDG-1)	Thr236	3i-loop	PKB (Akt)	HUVEC	Rac activation, cortical actin assembly, chemotaxis, angiogenesis	[53]
HEK293
CHO
Somatostatin sst_2A_	80% of phosphorylation on serine	3i-loop and/or C-terminal tail	ND	CHO	Contributes to desensitization	[40]
20% of phosphorylation on threonine	3i-loop and/or C-terminal tail	ND	CHO	Internalization and arrestin recruitment. Contributes to desensitization	

^a^Abbreviations: CHO, Chinese hamster ovary; COS-7, African green-monkey kidney cell line; DDT_1_MF2, a hamster vas deferens smooth-muscle cell line; EDG-1, endothelial differentiation gene 1; Grb2, growth-factor receptor-bound protein 2; HEK, human embryonic kidney cell; HUVEC, human umbilical-vein endothelial cells; i-loop, intracellular loop; IGF-1, insulin-like growth factor-1; JNK, c-Jun N-terminal; ND, not determined.

## Box 1 Model for differential receptor phosphorylation encoding differential signalling outcomes

Figure I depicts a single GPCR subtype that is able to be phosphorylated by three different protein kinases at distinct sites on the C-terminal tail and third intracellular loop. These hypothetical protein kinases are shown to be employed in different combinations resulting in different phosphorylation profiles or phosphorylation signatures.

The phosphorylation signature translates to a code (the phosphorylation code) that directs the signalling outcome of the receptor. In this process, GPCR phosphorylation is a flexible regulatory mechanism that can be tailored to suit the signalling requirements of a receptor in a given cell type. Thus, the same receptor subtype expressed in three different tissues (labelled tissues A–C in Figure I) can show different signalling outcomes based on differential receptor phosphorylation.
